# A Method of Constructing Measurement Matrix for Compressed Sensing by Chebyshev Chaotic Sequence

**DOI:** 10.3390/e22101085

**Published:** 2020-09-26

**Authors:** Renjie Yi, Chen Cui, Yingjie Miao, Biao Wu

**Affiliations:** 1 College of Electronic Countermeasure, National University of Defense Technology, Hefei 230000, China; kycuichen@163.com (C.C.); MiaoYJ_email@163.com (Y.M.); 2 Huayin Ordnance Test Center, Weinan 714000, China; wbuiao@163.com

**Keywords:** compressed sensing, measurement matrix, Chebyshev chaotic sequence, RIP

## Abstract

In this paper, the problem of constructing the measurement matrix in compressed sensing is addressed. In compressed sensing, constructing a measurement matrix of good performance and easy hardware implementation is of interest. It has been recently shown that the measurement matrices constructed by Logistic or Tent chaotic sequences satisfy the restricted isometric property (RIP) with a certain probability and are easy to be implemented in the physical electric circuit. However, a large sample distance that means large resources consumption is required to obtain uncorrelated samples from these sequences in the construction. To solve this problem, we propose a method of constructing the measurement matrix by the Chebyshev chaotic sequence. The method effectively reduces the sample distance and the proposed measurement matrix is proved to satisfy the RIP with high probability on the assumption that the sampled elements are statistically independent. Simulation results show that the proposed measurement matrix has comparable reconstruction performance to that of the existing chaotic matrices for compressed sensing.

## 1. Introduction

Compressed sensing (CS) [1] samples the signal at a rate far lower than the Nyquist rate by utilizing the signal’s sparsity. The original signal can be reconstructed from the sampled data by adopting corresponding reconstruction methods. As a new way of signal processing, CS reduces the amount of sampled data and the space of storage, which would significantly decrease the hardware complexity. CS has a broad application prospect in medical imaging [2], wideband spectrum sensing [3], dynamic mode decomposition [4], etc.

CS projects a high-dimensional *K*-sparse signal $x\in R^{N\times 1}$ into a low-dimensional space through the measurement matrix $\Phi \in R^{M\times N}$ (M<N) and gets a set of incomplete measurements $y\in R^{M\times 1}$ obeying the linear model

(1) y=Φx

Equation (1) is an underdetermined equation which usually has an infinite number of solutions. However, with prior information on the signal sparsity and a condition imposed on Φ, x can be exactly reconstructed by solving the $l_{1}$ minimization problem [5]. The most commonly used condition of Φ is the restricted isometric property (RIP) [6].

**Definition** **1.** *RIP: For any K-sparse vector x, if there is always a constant δ∈(0,1) that makes*

(2) $$(1-\delta ){\left\|x\right\|}_{2}^{2}\leq {\left\|\Phi x\right\|}_{2}^{2}\leq (1+\delta ){\left\|x\right\|}_{2}^{2}$$

*then Φ is said to satisfy the K-order RIP with*δ.

The minimum of all constants satisfying inequality (2) is referred to as the restricted isometry constant (RIC) $\delta_{K}$. Candès and Tao showed that exact reconstruction could be achieved from certain measurements provided Φ satisfies the RIP.

Constructing a proper measurement matrix is an essential issue in CS. The measurement matrix has significant influences not only on the reconstruction performance but also on the complexity of hardware implementation. The measurement matrices can be divided into random ones and deterministic ones. Gaussian random matrix [7] and Bernoulli random matrix [8] are frequently used because they satisfy the RIP and are uncorrelated with most sparse domains. They guarantee good reconstruction performance but bring a specific challenge to hardware such as the requirement for storage space and system design [9]. On the contrary, deterministic measurement matrices have the advantages not only of economic storage space but also of convenience in engineering design. Nevertheless, the commonly used deterministic measurement matrices such as deterministic polynomial matrix [10] and Fourier matrix [11] are correlated with certain sparse domains, resulting in a restriction on practical application.

For solving the problems, the Logistic chaotic sequence is employed in constructing the measurement matrix—called the Logistic chaotic measurement matrix—in [12]. With the natural gifted pseudo-random property of a chaotic system, the Logistic chaotic measurement matrix possesses the advantages of random matrices and overcomes the shortcoming of the above deterministic matrices. This kind of matrix is respectively used for secure data collection in wireless sensor networks in [13] and speech signal compression in [14]. In [15], the Tent chaotic sequence is employed in constructing the measurement matrix. In [16], Zhou and Jing construct the measurement matrix with a composite chaotic sequence generated by combining Logistic and Tent chaos. Compared with the Gaussian random matrix, the chaotic measurement matrices have lower hardware complexities and better reconstruction performances. However, large sample distances (at least 15 [17,18]) are required to obtain uncorrelated samples from the above chaotic sequences in chaotic measurement matrix construction. Numerous useless data will be generated when the measurement matrix is large-scale, which results in the waste of system resources. In [19], the Chebyshev chaotic sequence is transformed into a new sequence of elements obeying Gaussian distribution. The new sequence is employed in constructing the measurement matrix that satisfies the RIP with high probability. This method avoids sampling the chaotic sequence, but the measurement matrix does not significantly improve the reconstruction performance.

In this paper, we propose a method of constructing the measurement matrix by the Chebyshev chaotic sequence. The primary contributions are twofold:

- We analyze the high-order correlations among the elements sampled from the Chebyshev chaotic sequence.
- We use the sampled elements to construct a measurement matrix, termed the Chebyshev chaotic measurement matrix. Based on the assumption that the elements are statistically independent, we prove that the Chebyshev chaotic measurement matrix satisfies the RIP with high probability.

The remainder of this paper is organized as follows. In Section 2, we describe the expression of the Chebyshev chaotic sequence and analyze the high-order correlations among elements sampled from the Chebyshev chaotic sequence. In Section 3, we present the construction method of the Chebyshev chaotic measurement matrix and analyze its probability of satisfying the RIP. In Section 4, simulations are carried out to verify the effectiveness of the Chebyshev chaotic matrix. In the end, the conclusion is drawn.

## 2. Chebyshev Chaotic Sequence and Sample Distance

### 2.1. Chebyshev Chaotic Sequence

Chaotic systems generate deterministic sequences by recursive methods. The sequence naturally enjoys certain properties that greatly resemble what we perceive as randomness. Since the sequence is reproducible and passes tests of randomness, it is often used to generate pseudo-random numbers [20]. Chebyshev chaos is a typical nonlinear dynamic chaos. Its one-dimensional expression is written as

(3) $$x_{n+1}=\cos\left(q\cdot \arccos x_{n}\right)\;\;x_{n}\in \left[-1,1\right]$$

where q (≥2) denotes the order number of Chebyshev chaos. With the initial value $x_{0}$, the Chebyshev chaotic sequence is produced by applying Equation (3) recursively. On its excellent randomness, sensitivity to initial values, spatial ergodicity, and easy implementation in physical electric circuits [21], Chebyshev chaos is widely valued.

Based on the fact that some regions of the state space are visited more frequently than others by the Chebyshev chaotic sequence, it is possible to associate an invariant probability density function, denoted ρ(x), to a chaotic attractor. ρ(x) is as follows [22]:

(4) $$\rho (x)=\{\begin{array}{ccc} \frac{1}{\pi \sqrt{1-x^{2}}} & & x\in \left(-1,1\right)\\ 0 & & else \end{array}$$

From Equation (4), we have

(5) $$E(X_{n}^{t})=\left\{ \begin{array}{lll} 2^{-t}C_{t}^{t/2} & & t\;=\;0,2,4\cdots \\ 0 & & t\;=\;1,3,5\cdots \end{array}\right.$$

where $C_{t}^{t/2}$ denotes the number of t/2 subsets of a t-element set.

### 2.2. The Internal Randomness of Chebyshev Chaos

**Figure 1:**
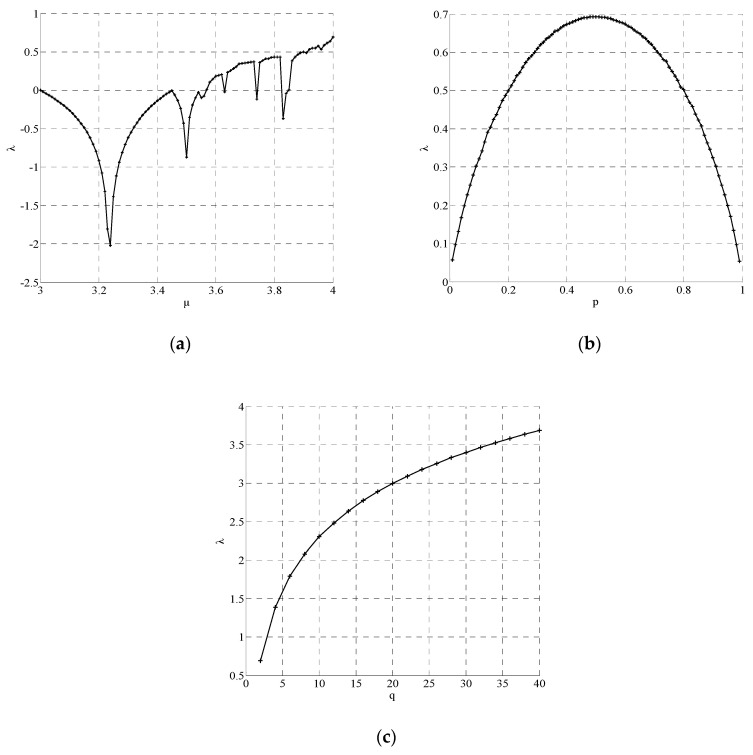
The change tendency of λ with system parameters: (**a**) Logistic; (**b**) Tent; (**c**) Chebyshev.

Internal randomness is one of the main characteristics of chaos. The chaos system is stable on the whole, but it will show regional instability due to the internal randomness. Regional instability is manifested in the sensitivity to initial conditions. The stronger the sensitivity, the stronger the internal randomness. The Lyapunov exponent [23] is a quantitative description of the sensitivity, which characterizes the average rate of divergence between the adjacent trajectories. The Lyapunov exponent expression of the one-dimensional discrete mapping system $x_{n+1}=F\left(x_{n}\right)$ is written as

(6) $$\lambda =\underset{n\to \infty }{\lim}\frac{1}{n}\sum_{j=0}^{n-1}\ln\left|F^{\prime }\left(x_{j}\right)\right|$$

where $F^{\prime }\left(x_{j}\right)$ denotes the derivative of $F\left(x_{j}\right)$ with respect to $x_{j}$. The trajectories will gradually close up until reclosing when λ≤0 and diverge when λ>0. A system with λ>0 is defined to be chaotic. The bigger the λ, the stronger the internal randomness. Set $x_{0}=0.5$, n=10000, the change tendency of λ with system parameters in different chaos is shown in Figure 1. The single control parameter is denoted as μ in Logistic chaos and p in Tent chaos.

As can be seen from Figure 1, in Logistic chaos, when μ is 4, λ reaches the maximum value of 0.69. In Tent chaos, λ reaches the maximum value of 0.69 when p is 0.5. In Chebyshev chaos, λ is 0.69 when q=2 and λ increases with q. Therefore, when q is greater than 2, the Lyapunov exponent of Chebyshev chaos is larger than that of Logistic chaos and Tent chaos, which means stronger internal randomness. Considering that the hardware complexity increases exponentially over q, we set q=8 so that Chebyshev chaos has a good balance between randomness and hardware implementation.

### 2.3. Statistical Property and Sample Distance

Generally, the elements in the measurement matrix need to be independent of each other. However, the elements in chaotic sequences do not meet the requirements. Yu et al. [12] measured the independence between the elements sampled from the Logistic chaotic sequence through the high-order correlations. The elements are considered approximately independent if ideal high-order correlations are available. These “independent” elements are used to construct the chaotic measurement matrix and good reconstruction performance is obtained. To construct the measurement matrix with the Chebyshev chaotic sequence, the primary issue is how to get independent elements from the sequence. Inspired by [12], we measure the independence through the high-order correlations with a certain sample distance and have the following theorem.

**Theorem** **1.** *Denote*$X=\left\{x_{n},x_{n+1}\cdots x_{n+k}\cdots \right\}$*as the sequence generated by Equation (3), and integer*d*as the sample distance. When*q*(*≥2*) is even, for an arbitrary positive integer*$m_{0},\;m_{1}<q^{d}$, *it has*

(7) $$E\left(x_{n}^{m_{0}}x_{n+d}^{m_{1}}\right)=E\left(x_{n}^{m_{0}}\right)E\left(x_{n+d}^{m_{1}}\right)$$

**Proof.**  See Appendix A.

By setting d=5 and q=8, we have $E\left(x_{n}^{m_{0}}x_{n+d}^{m_{1}}\right)=E\left(x_{n}^{m_{0}}\right)E\left(x_{n+d}^{m_{1}}\right)$ for all $m_{0},\;m_{1}<32768$. In [12], the elements sampled from the Logistic chaotic sequence share the same high-order correlations with d=15 and are considered approximately independent. In [17,18], the independence test algorithms are applied to determine the sample distance of the Logistic and Tent chaotic sequences. The test procedures indicate that the sampled elements are statistically independent when d≥15.

**Figure 2:**
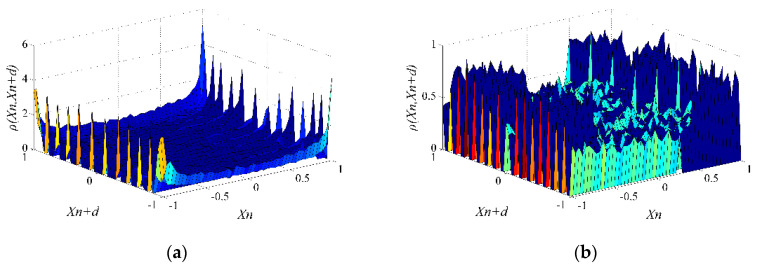

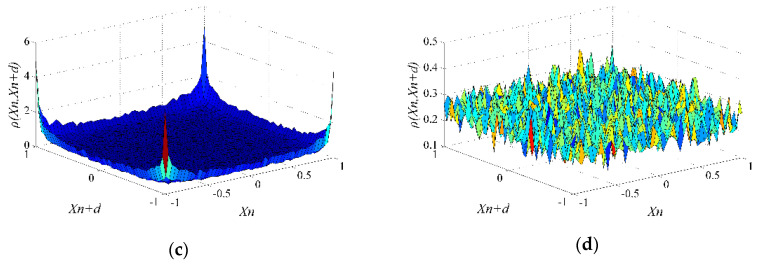
Logistic and Tent chaotic sequences. (**a**) Logistic d=5; (**b**) Tent d=5; (**c**) Logistic d=15; (**d**) Tent d=15.

**Figure 3:**
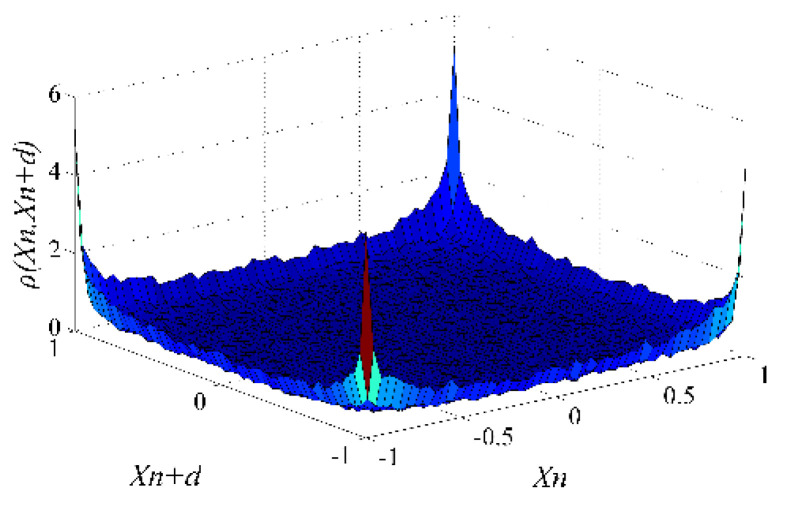
$\rho \left(x_{n},x_{n+d}\right)$ in the 8-order Chebyshev chaotic sequence with d=5.

Figure 2 and Figure 3 illustrate the joint probability densities of $x_{n}$ and $x_{n+d}$, and denote $\rho \left(x_{n},x_{n+d}\right)$, in Logistic, Tent, and 8-order Chebyshev chaotic sequences when d takes 5 and 15. The smoother the surface of $\rho \left(x_{n},x_{n+d}\right)$, the more uniform the distribution of $x_{n}$ and $x_{n+d}$, and the weaker the correlation between $x_{n}$ and $x_{n+d}$. In Figure 2a,b, the surfaces of $\rho \left(x_{n},x_{n+d}\right)$ in Logistic and Tent have large fluctuations when d=5, which means strong correlations between $x_{n}$ and $x_{n+d}$. In Figure 2c,d, the surfaces of $\rho \left(x_{n},x_{n+d}\right)$ in Logistic and Tent are relatively gentle, indicating a weak correlation between $x_{n}$ and $x_{n+d}$ when d=15. It can be seen intuitively from Figure 3 that the surface of $\rho \left(x_{n},x_{n+d}\right)$ in Chebyshev is almost the same as that in Figure 2c. These figures show that the elements sampled from the 8-order Chebyshev chaotic sequence with d=5 share the same correlations with those sampled from the Logistic chaotic sequence with d=15.

Therefore, to guarantee a very small correlation between the sampled elements, the sample distance required by the 8-order Chebyshev chaotic sequence is significantly smaller than those required by the Logistic and Tent chaotic sequences. According to Section 2.2, the internal randomness of 8-order Chebyshev chaos is stronger than that of Logistic and Tent chaos. The increase in internal randomness leads to the decrease in the correlation between $x_{n}$ and $x_{n+d}$, and the decrease in the sample distance.

## 3. Construction of Chebyshev Chaotic Measurement Matrix and RIP Analysis

### 3.1. Construction of Chebyshev Chaotic Measurement Matrix

Denote $Z=\left\{z_{1},z_{2}\cdots z_{n}\right\}$ as the sequence extracted from X with sample distance d. We use Z to construct a Chebyshev chaotic measurement matrix as shown in **Algorithm 1**.

**Algorithm 1.** The method of constructing the Chebyshev chaotic measurement matrix.
**Input: the number of rows M, the number of columns N, order q, initial value $x_{0}$.**

**Output: measurement matrix**
Φ

**1.**
Determine the sample distance d;

**2.**
Generate the Chebyshev chaotic sequence X of length 1+(MN−1)d;
**3.**
Sample X with d and get $Z=\left\{z_{1},z_{2}\cdots z_{MN}\right\}$;
**4.**
Use Z to construct a Chebyshev chaotic measurement matrix as
$\Phi =\sqrt{\frac{2}{M}}\left(\begin{array}{cccc} z_{1} & z_{M+1} & \cdots & z_{\left(N-1\right)M+1}\\ z_{2} & z_{M+2} & \cdots & z_{\left(N-1\right)M+2}\\ \vdots & \vdots & \ddots & \vdots \\ z_{M} & z_{2M} & \cdots & z_{MN} \end{array}\right)$
**5.**
Return measurement matrix Φ


In step 4, $\sqrt{2/M}$ is the normalization factor. When the order number, initial value, and sample distance are set, Φ is determined.

### 3.2. RIP Analysis

The RIP is a sufficient condition on the measurement matrix to recover guarantee. However, certifying the RIP is NP-hard, so we analyze the performance of the measurement matrix Φ by calculating the probability that Φ satisfies the RIP instead.

When d=5, the adjacent elements of Z share the same high-order correlations with the elements sampled from the Logistic chaotic sequence with d=15. In addition, the test procedures show that the sampled elements of the Logistic chaotic sequence are statistically independent when d=15. Hence, we assume that the elements of Z are statistically independent. Based on this assumption, we calculate the minimum of the probability that our proposed measurement matrix satisfies the RIP and show that the minimum is close to 1 provided some parameters are suitably valued. The main result is shown in Theorem 2.

**Theorem** **2.** *TheChebyshev chaotic measurement matrix*$\Phi \in R^{M\times N}$*constructed by Algorithm 1 satisfies the K-order RIP with high probability provided*K≤O(M/log(N/K)).

Before proving Theorem 2, we briefly summarize some useful notations. Denote $\left(l_{1},l_{2}\cdots l_{K}\right)$ as the position of non-zero elements in x and $u={\left(u_{1},u_{2}\cdots u_{K}\right)}^{T}$ as the normalized vector composed of non-zero elements in x. $\Phi_{K}$ is a submatrix composed of Φ that only contains columns indexed by $\left(l_{1},l_{2}\cdots l_{K}\right)$. Rewrite $\Phi_{K}$ as $\Phi_{K}=\sqrt{1/M}{\left(\varphi_{1},\varphi_{2}\cdots \varphi_{M}\right)}^{T}$, where $\varphi_{m}^{T}=\left(v_{1},v_{2}\cdots v_{K}\right)$ denotes a vector formed by multiplying the elements positioned at $\left(l_{1},l_{2}\cdots l_{K}\right)$ in the m th row of Φ by $\sqrt{2}$, and m=1,2⋯M. Let $S={\left\|s\right\|}_{2}^{2}=\sum_{m=1}^{M}Q_{m}^{2}/M$, $s=\Phi_{K}u$, and $Q_{m}=\varphi_{m}^{T}u$. Denote $G_{g}=\left\{\left|{\left\|\Phi_{K}u\right\|}_{2}^{2}-1\right|\geq \delta \right\}$ as one complementary event of the condition in inequality (2), where $g=1,2\cdots C_{N}^{K}$. $G=\cup_{g=1}^{C_{N}^{K}}G_{g}$ is the union of all possible complementary events. The idea of proving Theorem 2 is as follows: First, calculate the probability of $G_{g}$, which is denoted as $P\left(G_{g}\right)$. Then, compute the probability of G, denoted as P(G). Finally, the probability of Φ satisfying the RIP is $P_{RIP}=1-P\left(G\right)$.

From the definition of $G_{g}$, we have

(8) $$P\left(G_{g}\right)\leq P\left(S\geq 1+\delta \right)+P\left(S\leq 1-\delta \right)$$

where $P\left(S\geq 1+\delta \right)=P\left[\sum_{m=1}^{M}Q_{m}^{2}\geq \left(1+\delta \right)M\right]$ and $P\left(S\geq 1+\delta \right)=\;P\left\{\exp\left(\alpha \sum_{m=1}^{M}Q_{m}^{2}\right)\geq \exp\left[\alpha \left(1+\delta \right)M\right]\right\}$ holds for any α>0. According to Markov inequality, the following inequality holds

(9) $$P\left(S\geq 1+\delta \right)\leq \;E\left[\exp\left(\alpha \sum_{m=1}^{M}Q_{m}^{2}\right)\right]\exp\left[-\alpha \left(1+\delta \right)M\right]$$

Noting that the elements of $\Phi_{K}$ are statistically independent of each other, it is evident that $Q_{h}$ and $Q_{m}$ are independent of each other for l≠m, l,m=1,2⋯M. With $E\left[\exp\left(\alpha Q_{j}^{2}\right)\right]=E\left[\exp\left(\alpha Q_{h}^{2}\right)\right]$, the inequality can be rewritten as

(10) $$P\left(S\geq 1+\delta \right)\leq \;{\left\{E\left[\exp\left(\alpha Q_{m}^{2}\right)\right]\right\}}^{M}\exp\left[-\alpha \left(1+\delta \right)M\right]$$

In the same way, we have

(11) $$P\left(S\leq 1-\delta \right)\leq \;{\left\{E\left[\exp\left(-\alpha Q_{m}^{2}\right)\right]\right\}}^{M}\exp\left[\alpha \left(1-\delta \right)M\right]$$

By expanding $\exp\left(-\alpha Q_{j}^{2}\right)$ into the form of a second-order Lagrange remainder, $\exp\left(-\alpha Q_{m}^{2}\right)=\sum_{t=0}^{2}{\left(-\alpha Q_{m}^{2}\right)}^{t}/t!+R_{2}$ holds, where $R_{2}$ is the Lagrange remainder. It is straightforward to verify $R_{2}\leq 0$. Accordingly, we have $\exp\left(-\alpha Q_{m}^{2}\right)\leq \sum_{t=0}^{2}{\left(-\alpha Q_{m}^{2}\right)}^{t}/t!$ and

(12) $$P\left(S\leq 1-\delta \right)\leq \;{\left[E\left(1-\alpha Q_{m}^{2}+\alpha^{2}Q_{m}^{4}/2\right)\right]}^{M}\exp\left[\alpha \left(1-\delta \right)M\right]$$

To calculate the probabilities in inequalities (10) and (12), let us introduce some useful lemmas.

**Lemma** **1.** *Denote* $r_{1}$ *, $r_{2}$ i.i.d. as random variables having a probability distribution like Equation (4). For any real numbers a, b, let $c=\sqrt{\left(a^{2}+b^{2}\right)/2}$, then for all T∈R and t∈N, we have*

(13) $$E\left[{\left(T+ar_{1}+br_{2}\right)}^{2t}\right]\leq E\left[{\left(T+cr_{1}+cr_{2}\right)}^{2t}\right]$$

**Proof.**  See Appendix B.

**Lemma** **2.** *Let* $w=\frac{1}{\sqrt{K}}{\left(1,1\cdots 1\right)}^{T}$ *be a unit vector. For arbitrary* t∈N *and m=1,2⋯M, we have*

(14) $$E\left[Q_{m}^{2t}\right]\leq E\left[{\left(\varphi_{m}^{T}w\right)}^{2t}\right]$$

**Proof.**  See Appendix C.

**Lemma** **3.** *Let*T∼N(0,1)*and*$\varphi_{m}={\left(v_{1},v_{2}\cdots v_{K}\right)}^{T}$*. Then, for arbitrary*t∈N, we have

(15) $$E\left[{\left(\varphi_{m}^{T}\omega \right)}^{2t}\right]\leq E\left(T^{2t}\right)$$

**Proof.**  See Appendix D.

Recall T∼N(0,1). For arbitrary α∈[0,1/2), the Taylor series expansion of $E\left[\exp\left(\alpha T^{2}\right)\right]$ is $\sum_{t=0}^{\infty }{\left(\alpha \right)}^{t}E\left(T^{2t}\right)/t!$. In the same way, $E\left[\exp\left(\alpha Q_{m}^{2}\right)\right]=\sum_{t=0}^{\infty }{\left(\alpha \right)}^{t}E\left(Q_{m}^{2t}\right)/t!$. Applying Lemma 2 and 3 gives $E\left(Q_{m}^{2t}\right)\leq E\left(T^{2t}\right)$. Hence, $E\left[\exp\left(\alpha Q_{m}^{2t}\right)\right]\leq E\left[\exp\left(\alpha T^{2}\right)\right]=1/\sqrt{1-2\alpha }$ holds.

Now, we calculate the probabilities in inequalities (10) and (12). Let α=δ/[2(1+δ)], we get

(16) $$\begin{array}{ll} P\left(S\geq 1+\delta \right) & \leq \;{\left(1/\sqrt{1-2\alpha }\right)}^{M}\exp\left[-\alpha \left(1+\delta \right)M\right]\\ & ={\left[\left(1+\delta \right)\exp\left(-\delta \right)\right]}^{M/2}\\ & \;<\exp\left[-\frac{M}{2}\left(\frac{\delta^{2}}{2}-\frac{\delta^{3}}{3}\right)\right] \end{array}$$

As $E\left(Q_{m}^{2}\right)=1$ and $E\left(Q_{m}^{4}\right)\leq E\left(T^{4}\right)=3$, we have

(17) $$\begin{array}{ll} P\left(S\leq 1-\delta \right) & \leq \;{\left(1-\alpha +3\alpha^{2}/2\right)}^{M}\exp\left[\alpha \left(1-\delta \right)M\right]\\ & ={\left[1-\frac{\delta }{2\left(1+\delta \right)}+\frac{3\delta^{2}}{8{\left(1+\delta \right)}^{2}}\right]}^{M/2}\exp\left[\frac{\delta \left(1-\delta \right)}{2\left(1+\delta \right)}M\right]\\ & \;<\exp\left[-\frac{M}{2}\left(\frac{\delta^{2}}{2}-\frac{\delta^{3}}{3}\right)\right] \end{array}$$

Therefore,

(18) $$P\left(G_{g}\right)\leq \;2\exp\left[-\frac{M}{2}\left(\frac{\delta^{2}}{2}-\frac{\delta^{3}}{3}\right)\right]$$

According to Boole’s inequality, we get

(19) $$\begin{array}{ll} P\left(G\right) & \leq \;\sum_{g=1}^{C_{N}^{K}}P\left(G_{g}\right)\\ & \leq 2C_{N}^{K}\exp\left[-\frac{M}{2}\left(\frac{\delta^{2}}{2}-\frac{\delta^{3}}{3}\right)\right]\\ & \leq 2{\left(\frac{eN}{K}\right)}^{K}\exp\left[-\frac{M}{2}\left(\frac{\delta^{2}}{2}-\frac{\delta^{3}}{3}\right)\right] \end{array}$$

Let $C_{1}>0$ and $C_{1}M\geq K\log\left(N/K\right)$, we have

(20) $$P\left(G\right)\leq 2\exp\left[-\frac{M}{2}\left(\frac{\delta^{2}}{2}-\frac{\delta^{3}}{3}\right)+C_{1}M+\frac{C_{1}M}{\log\left(N/K\right)}\right]$$

Let $C_{2}\leq \frac{1}{2}\left(\frac{\delta^{2}}{2}-\frac{\delta^{3}}{3}\right)-C_{1}\left(1+\frac{1}{\log\left(N/K\right)}\right)$. Then, Φ satisfies the RIP with a probability of

(21) $$P_{RIP}\geq 1-2\exp\left(-C_{2}M\right)$$

Indeed, choosing $C_{1}$ as sufficiently small, we always have $C_{2}>0$ and high $P_{RIP}$. For instance, let N=512 and K=5, the probability of Φ satisfying the *K*-order RIP with $\delta_{c}=0.9$ is no less than 95% when $C_{1}=0.0589$.

## 4. Results and Discussion

When a measurement matrix is applied in CS, it is always expected to lead to good reconstruction performance. In this section, we examine the performance of the Chebyshev chaotic measurement matrix and compare it with Gaussian random matrix and the well-established similar matrices in [12,15,16,19] by presenting the empirical results obtained from a series of CS reconstruction simulations. Each matrix is denoted as Propose, Gaussian, Logistic, Tent, Composite, and the matrix in [19] for convenience. The test includes the following steps. First, generate the synthetic sparse signals x and construct the measurement matrix Φ. Then, obtain the measurement vector y by y=Φx. Last, reconstruct the signals by approximating the solution of $x_{e}=\arg\min{\left\|x\right\|}_{o}\;s.t.\;y=\Phi x$.

x adopted throughout this section only contains *K* non-zero entries with length N=120. The locations and amplitudes of the peaks are subject to Gaussian distribution. The proposed measurement matrix in this paper is constructed by the method shown in Algorithm 1. The orthogonal matching pursuit (OMP) [24] algorithm is selected as a minimization approach which iteratively builds up an approximation of x. The system parameters in Logistic and Tent chaos are 4 and 0.5, respectively, and the order of Chebyshev chaos is 8. The sample distance is set as d=15 in Logistic, Tent, and Composite, and d=5 in Propose. Each experiment is performed for 1000 random sparse ensembles. The initial value $x_{0}$ is randomly set for each ensemble and the performance is averaged over sequences with different initial values. Denote $\varepsilon ={\left\|x_{e}-x\right\|}_{2}^{}/{\left\|x\right\|}_{2}^{}$ as the reconstruction error. The reconstruction is identified as a success, namely exact reconstruction, provided $\varepsilon \leq 10^{-6}$. Denote $P_{suc}$ as the percentage of successful reconstruction.

**Case** **1.** Comparison of the percentage of exact reconstruction in the noiseless case $P_{suc}$.

In this case, we conduct two separate CS experiments, first by fixing M=40 and varying K from 2 to 14, and second by fixing K=8 and varying M from 20 to 60.

**Figure 4:**
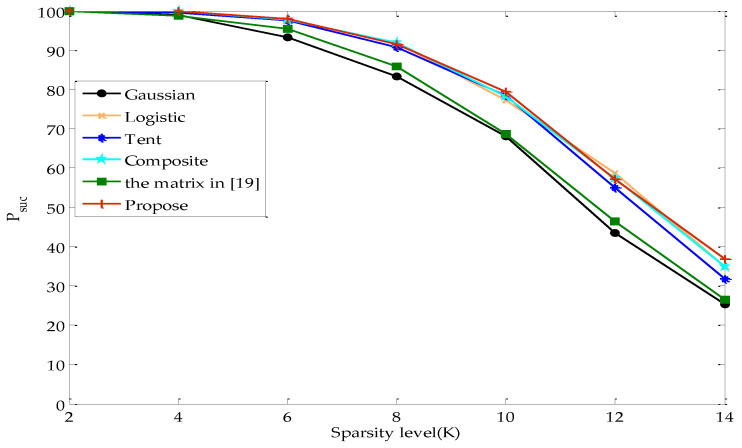
The change tendency of $P_{suc}$ with *K* while M=40 in the noiseless case.

Figure 4 illustrates the change tendency of $P_{suc}$ with argument K while M=40 in the noiseless case. The figure shows that $P_{suc}$ decreases with the increase in *K*. From inequalities (20) and (21), it can be seen that the upper bound of P(G) increases and the lower bound of $P_{RIP}$ decreases when *K* increases. The probability of the measurement matrix satisfying the RIP is reduced, which makes it against the exact reconstruction.

**Figure 5:**
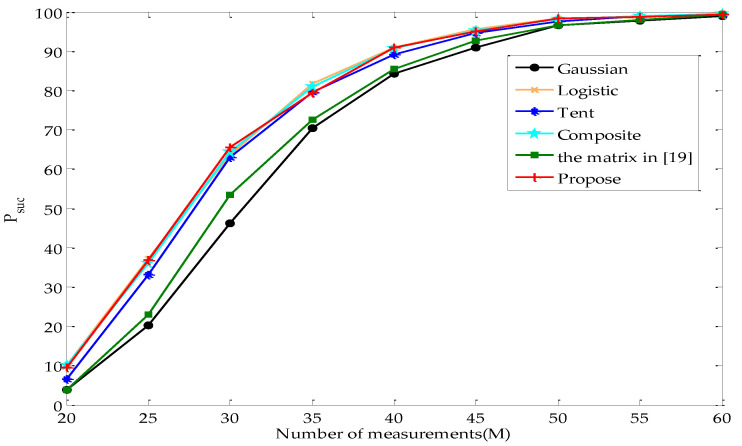
The change tendency of $P_{suc}$ with M while K=8 in the noiseless case.

Figure 5 illustrates the change tendency of $P_{suc}$ with argument M while K=8 in the noiseless case. As can be seen from the figure, $P_{suc}$ increases with M. According to inequalities (20) and (21), P(G) decreases and the lower bound of $P_{RIP}$ increases when M increases. The increase in the probability of the measurement matrix satisfying the RIP is beneficial to the exact reconstruction.

Figure 4 and Figure 5 reveal that the percentage of the exact reconstruction of the proposed measurement matrix is significantly higher than that of Gaussian and the matrix in [19], slightly higher than that of Tent, and almost the same as that of Logistic and Composite.

**Case** **2.** Comparison of the reconstruction error in the noisy case.

**Figure 6:**
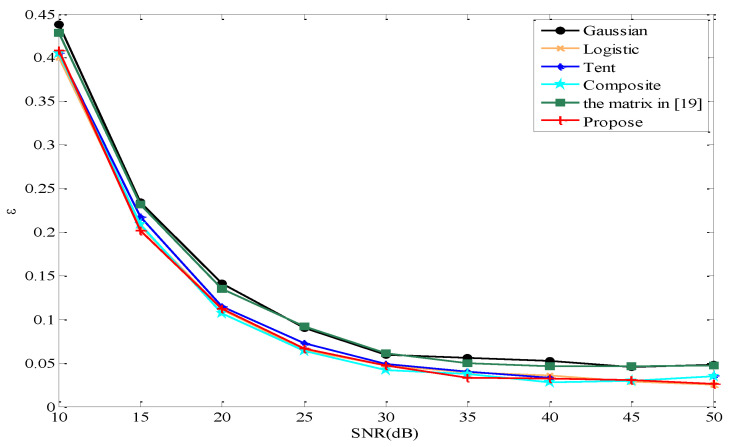
The change tendency of ε with SNR while M=40 and K=8.

In this case, we consider the noisy model y=Φx+v, where v is the vector of additive Gaussian noise with zero means. We conduct the CS experiment by fixing M=40, K=8, and varying the signal to noise ratio (SNR) from 10 to 50. It can be seen from Figure 6 that the errors decrease with the increase in the SNR, and the error of the proposed measurement matrix is smaller than that of Gaussian and the matrix in [19], slightly smaller than that of Tent, and almost the same as Logistic and Composite.

When noise is included in the measurements, the reconstruction errors increase greatly. At this time, reconstruction algorithms with anti-noise ability can be used, such as the adaptive iterative threshold (AIT) algorithm and entropy minimization-based matching pursuit (EMP) algorithm which can be found in references [25,26] for details.

As shown in the simulations, when the Chebyshev chaotic matrix is applied in CS, a good reconstruction performance is obtained. This coincides with the theoretical results obtained in the previous subsection where the Chebyshev chaotic matrix satisfies the RIP with high probability. Recall from Lemma 4 that $E\left[\exp\left(\alpha Q_{m}^{2}\right)\right]\leq E\left[\exp\left(\alpha T^{2}\right)\right]$, where T∼N(0,1). It is clear that the maximums of P(S≥1+δ) and P(S≤1−δ) are achievable only if $Q_{m}∼N\left(0,1\right)$. That is to say, the lower boundary of the probability that the proposed measurement satisfies the RIP is no less than that of the Gaussian random matrix. As seen from the simulations, our proposed measurement matrix outperforms the Gaussian random matrix. Then, the results coincide with the theoretical analysis above.

As mentioned in the Introduction, Zhu et al. [19] transformed the Chebyshev chaotic sequence into a new sequence of elements obeying Gaussian distribution and applied the new sequence to construct the measurement matrix. In fact, the measurement matrix is similar to the Gaussian random matrix in terms of elements distribution. This coincides with the simulations that the reconstruction performance of OMP with the matrix in [19] is similar to that of OMP with the Gaussian random matrix. Accordingly, our proposed measurement matrix outperforms the matrix in [19].

The simulations reveal that by using the proposed measurement matrix, one can achieve the same reconstruction performance as that obtained using Logistic, Tent, and Composite. In fact, these chaotic measurement matrices share a similar independence among the elements and probability of satisfying the RIP. To construct those measurement matrices, MN uncorrelated samples need to be extracted from the chaotic sequences at certain sample distances. The length of the chaotic sequence is usually no less than MNd. The larger the sample distance, the longer the chaotic sequence, and the larger the resources consumption. Since the sample distance of the Chebyshev chaotic sequence is greatly reduced, the proposed measurement matrix can effectively reduce the consumption of system resources.

## 5. Conclusions

In this paper, we propose a method of constructing a measurement matrix for compressed sensing by the Chebyshev chaotic sequence. We first show that the elements sampled from the Chebyshev chaotic sequence with a small distance have very small correlations. Then, we use these sampled elements to construct the measurement matrix and prove that the matrix satisfies the RIP with high probability in detail. With the natural gifted pseudo-random property of a chaotic system, the proposed chaotic measurement matrix possesses the advantages of economic storage and convenience in engineering design. Moreover, the probability that the proposed measurement matrix satisfies the RIP is more likely to be higher than that of the Gaussian random matrix dose, which results in better reconstruction performance. Obviously, the proposed measurement matrix outperforms the Gaussian random one. Compared with the similar chaotic measurement matrices, the proposed measurement matrix effectively reduces the consumption of system resources without the loss of reconstruction performance. Therefore, our method outperforms the existing approaches in terms of practical applications.

## Appendix A

**Proof** **of** **Theorem** **1.** According to Equation (3), we have $x_{n+d}=\cos\left(q^{d}\cdot \arccos x_{n}\right)$. Then, the left side of Equation (7) can be expressed as

(A1) $$E\left(x_{n}^{m_{0}}x_{n+d}^{m_{1}}\right)=\int_{-1}^{1}\rho \left(x_{n}\right)x_{n}^{m_{0}}{\left[\cos\left(q^{d}\arccos x_{n}\right)\right]}^{m_{1}}dx_{n}$$

Denote $x_{n}=\cos\theta$, substituting Equation (4) into (A1), we obtain

(A2) $$E\left(x_{n}^{m_{0}}x_{n+d}^{m_{1}}\right)=\int_{0}^{\pi }\frac{1}{\pi }{\left(\cos\theta \right)}^{m_{0}}{\left[\cos\left(q^{d}\theta \right)\right]}^{m_{1}}d\theta$$

Since

$$\left\{ \begin{array}{l} \cos\theta =\left(e^{i\theta }+e^{-i\theta }\right)/2\\ \int_{0}^{\pi }\frac{1}{\pi }e^{i2\theta k}d\theta =\{\begin{array}{ccc} 0 & & k=1,2,3\cdots \\ 1 & & k=0 \end{array} \end{array}\right.$$

Equation (A2) can be rewritten as

(A3) $$E\left(x_{n}^{m_{0}}x_{n+d}^{m_{1}}\right)=\int_{0}^{\pi }2^{-\left(m_{0}+m_{1}\right)}\sum_{h=0}^{m_{0}}\sum_{j=0}^{m_{1}}C_{m_{0}}^{f}C_{m_{1}}^{j}\frac{1}{\pi }e^{i2\theta \left[h-m_{0}/2+q^{d}\left(j-m_{1}/2\right)\right]}d\theta$$

It can be seen from Equation (A3) that $E\left(x_{n}^{m_{0}}x_{n+d}^{m_{1}}\right)$ has non-zero values only when $j=m_{1}/2-\left(h-m_{0}/2\right)q^{-d}$, with $j\in \left(\left(m_{1}-1\right)/2,\left(m_{1}+1\right)/2\right)$. For all possible cases of $m_{0}$ and $m_{1}$, we do the following analysis:

(1) $m_{0}$ is odd. According to Equation (5), $E\left(x_{n}^{m_{0}}\right)$ is equal to 0 and then $E\left(x_{n}^{m_{0}}\right)E\left(x_{n+d}^{m_{1}}\right)=0$. We hypothesize that $E\left(x_{n}^{m_{0}}x_{n+d}^{m_{1}}\right)$ has non-zero values, which means there exists a positive integer j that satisfies $j=m_{1}/2-\left(h-m_{0}/2\right)q^{-d}$. Noting that $j\in \left(\left(m_{1}-1\right)/2,\left(m_{1}+1\right)/2\right)$, the only choice of j is $m_{1}/2$, where $m_{1}$ must be even. Accordingly, h must be equal to $m_{0}/2$. However, $h=m_{0}/2$ is not an integer when $m_{0}$ is odd, which contradicts that h must be an integer. Therefore, the hypothesis fails, that is, $E\left(x_{n}^{m_{0}}x_{n+d}^{m_{1}}\right)=0$ holds when $m_{0}$ is odd. In this case, $E\left(x_{n}^{m_{0}}x_{n+d}^{m_{1}}\right)=E\left(x_{n}^{m_{0}}\right)E\left(x_{n+d}^{m_{1}}\right)$ holds.

(2) $m_{0}$ is even. Two cases are discussed:

- $m_{1}$ is odd. According to Equation (5), $E\left(x_{n}^{m_{0}}\right)E\left(x_{n+d}^{m_{1}}\right)=0$ holds. We hypothesize that $E\left(x_{n}^{m_{0}}x_{n+d}^{m_{1}}\right)$ has non-zero values, which means there exists a positive integer j that satisfies $j=m_{1}/2-\left(h-m_{0}/2\right)q^{-d}$. When $m_{1}$ is odd, there is no integer j satisfying $j\in \left(\left(m_{1}-1\right)/2,\left(m_{1}+1\right)/2\right)$. Therefore, the hypothesis fails and $E\left(x_{n}^{m_{0}}x_{n+d}^{m_{1}}\right)=0$ holds. In this case, $E\left(x_{n}^{m_{0}}x_{n+d}^{m_{1}}\right)$ is equal to $E\left(x_{n}^{m_{0}}\right)E\left(x_{n+d}^{m_{1}}\right)$.
- $m_{1}$ is even. According to Equation (5), we have $E\left(x_{n}^{m_{0}}\right)E\left(x_{n+d}^{m_{1}}\right)=2^{-\left(m_{0}+m_{1}\right)}C_{m_{0}}^{m_{0}/2}C_{m_{1}}^{m_{1}/2}$. When $h=m_{0}/2$, there is an integer $j=m_{1}/2$ that makes $E\left(x_{n}^{m_{0}}x_{n+d}^{m_{1}}\right)$ be equal to $2^{-\left(m_{0}+m_{1}\right)}C_{m_{0}}^{m_{0}/2}C_{m_{1}}^{m_{1}/2}$. In this case, $E\left(x_{n}^{m_{0}}x_{n+d}^{m_{1}}\right)=E\left(x_{n}^{m_{0}}\right)E\left(x_{n+d}^{m_{1}}\right)$ holds.

In conclusion, the proof is completed. □

## Appendix B

**Proof** **of** **Lemma** **1.** Both sides of inequality (13) can be rewritten as

$$\{\begin{array}{c} E\left[{\left(R+ar_{1}+br_{2}\right)}^{2t}\right]=\sum_{i=0}^{2t}C_{2t}^{i}R^{i}E\left[{\left(ar_{1}+br_{2}\right)}^{2t-i}\right]\\ E\left[{\left(R+cr_{1}+cr_{2}\right)}^{2t}\right]=\sum_{i=0}^{2t}C_{2t}^{i}R^{i}E\left[{\left(cr_{1}+cr_{2}\right)}^{2t-i}\right] \end{array}$$

According to Equation (5), we have $E\left[{\left(ar_{1}+br_{2}\right)}^{2t-i}\right]=0$ and $E\left[{\left(cr_{1}+cr_{2}\right)}^{2t-i}\right]=0$ when i is odd. Obviously, $E\left[{\left(ar_{1}+br_{2}\right)}^{2t-i}\right]$ is equal to $E\left[{\left(cr_{1}+cr_{2}\right)}^{2t-i}\right]$. Now, we consider the problem of proving $E\left[{\left(ar_{1}+br_{2}\right)}^{2t-i}\right]\leq E\left[{\left(cr_{1}+cr_{2}\right)}^{2t-i}\right]$ when i is even. This is equivalent to proving

(A4) $$E\left[{\left(ar_{1}+br_{2}\right)}^{2t}\right]\leq E\left[{\left(cr_{1}+cr_{2}\right)}^{2t}\right]$$

for all t∈N.

It is easy to verify that inequality (A4) holds when t=0,1,2. The focus of the proof is on t>2. Since the odd-order moments of $r_{1}$ and $r_{2}$ are 0, the expansion of both sides in inequality (25) only have even-order moments whose coefficients are no less than 0. Hence, it is true if either a or b is positive or negative. Without the loss of generality, assume that a, b≥0. Then, we have to make sure that the following inequality

(A5) $$E\left[{\left(\frac{a}{\sqrt{2}c}r_{1}+\frac{b}{\sqrt{2}c}r_{2}\right)}^{2t}\right]\leq E\left[{\left(\frac{\sqrt{2}}{2}r_{1}+\frac{\sqrt{2}}{2}r_{2}\right)}^{2t}\right]$$

holds for t=3,4,5⋯ and a, b≥0.

On both sides of inequality (A5), the square sum of the coefficients of $r_{1}$ and $r_{2}$ is always 1. Let $a/\left(\sqrt{2}c\right)=\cos\theta$ and $b/\left(\sqrt{2}c\right)=\sin\theta$, where θ∈[0,π/2], inequality (A5) can be rewritten as

(A6) $$E\left[{\left(r_{1}\cos\theta +r_{2}\sin\theta \right)}^{2t}\right]\leq E\left[{\left(r_{1}\cos\frac{\pi }{4}+r_{2}\sin\frac{\pi }{4}\right)}^{2t}\right]$$

Therefore, the proof of inequality (A4) is equivalent to the proof of inequality (A6), that is, we need to prove that the maximum of $E\left[{\left(r_{1}\cos\theta +r_{2}\sin\theta \right)}^{2t}\right]$ occurs when θ is π/4. Let $f\left(\theta \right)=E\left[{\left(r_{1}\cos\theta +r_{2}\sin\theta \right)}^{2t}\right]$, the derivative of f(θ) with respect to θ is

(A7) $$f^{\prime }\left(\theta \right)=E\left[2t{\left(r_{1}\cos\theta +r_{2}\sin\theta \right)}^{2t-1}\left(r_{2}\cos\theta -r_{1}\sin\theta \right)\right]$$

Since $E\left[{\left(r_{1}\cos\theta +r_{2}\sin\theta \right)}^{2t-1}r_{1}\right]=\sum_{h=0}^{2t-1}C_{2t-1}^{h}{\left(\cos\theta \right)}^{h}{\left(\sin\theta \right)}^{2t-1-h}E\left(r_{1}{}^{h+1}r_{2}{}^{2t-1-h}\right)$ and $E\left(r_{1}{}^{h+1}r_{2}{}^{2t-1-h}\right)=E\left(r_{2}{}^{h+1}r_{1}{}^{2t-1-h}\right)$, we have

(A8) $$E\left[{\left(r_{1}\cos\theta +r_{2}\sin\theta \right)}^{2t-1}r_{1}\right]=E\left[{\left(r_{2}\cos\theta +r_{1}\sin\theta \right)}^{2t-1}r_{2}\right]$$

Substituting Equation (A8) into Equation (A7), we obtain

$$\begin{array}{ll} f^{\prime }\left(\theta \right) & =2t\cdot E\left[{\left(r_{1}\cos\theta +r_{2}\sin\theta \right)}^{2t-1}r_{2}\cos\theta -{\left(r_{2}\cos\theta +r_{1}\sin\theta \right)}^{2t-1}r_{2}\sin\theta \right]\\ & =2t\cdot \sum_{h=0}^{2t-1}C_{2t-1}^{h}\left\{\left[{\left(\cos\theta \right)}^{h+1}{\left(\sin\theta \right)}^{2t-1-h}-{\left(\sin\theta \right)}^{h+1}{\left(\cos\theta \right)}^{2t-1-h}\right]E\left(r_{1}{}^{h}r_{2}{}^{2t-h}\right)\right\} \end{array}$$

Let $A(h)=C_{2t-1}^{h}\left\{\left[{\left(\cos\theta \right)}^{h+1}{\left(\sin\theta \right)}^{2t-1-h}-{\left(\sin\theta \right)}^{h+1}{\left(\cos\theta \right)}^{2t-1-h}\right]E\left(r_{1}{}^{h}r_{2}{}^{2t-h}\right)\right\}$. Note that A(t−1)=A(2t−1)=0, $f^{\prime }\left(\theta \right)$ can be rewritten as

(A9) $$\begin{array}{ll} f^{\prime }\left(\theta \right) & =2t\left[\sum_{h=0}^{t-2}A(h)+A(t-1)+\sum_{h=t}^{2t-2}A(h)+A(2t-1)\right]\\ & =2t\left[\sum_{h=0}^{t-2}A(h)+\sum_{h=t}^{2t-2}A(h)\right]\\ & =\;2t\sum_{h=0}^{t-2}\left[A(h)+A(2t-2-h)\right] \end{array}$$

With further simplifications, we get

$$f^{\prime }\left(\theta \right)=2t\sum_{h=0}^{t-2}\left\{\left[{\left(\cos\theta \right)}^{2t-1-h}{\left(\sin\theta \right)}^{h+1}-{\left(\sin\theta \right)}^{2t-1-h}{\left(\cos\theta \right)}^{h+1}\right]B_{h}\right\}$$

where $B_{h}=C_{2t-1}^{h+1}E\left(r_{1}{}^{2t-h-2}r_{2}{}^{h+2}\right)-C_{2t-1}^{h}E\left(r_{1}{}^{h}r_{2}{}^{2t-h}\right)$ and h∈[0,t−2]. When h is odd, it is straightforward to show that $B_{h}=0$. When h is even, according to Equation (5), $B_{h}$ can be expressed as

$$\begin{array}{ll} B_{h} & =2^{-2t}\left\{\frac{\left[\left(2t-1\right)!\right]\left(h+2\right)}{{\left[\left(\frac{2t-h-2}{2}\right)!\right]}^{2}{\left[\left(\frac{h+2}{2}\right)!\right]}^{2}}-\frac{\left[\left(2t-1\right)!\right]\left(2t-h\right)}{{\left[\left(\frac{2t-h}{2}\right)!\right]}^{2}{\left[\left(\frac{h}{2}\right)!\right]}^{2}}\right\}\\ & =\frac{4\cdot 2^{-2t}\left[\left(2t-1\right)!\right]}{{\left[\left(\frac{2t-h-2}{2}\right)!\right]}^{2}{\left[\left(\frac{h}{2}\right)!\right]}^{2}}\left(\frac{1}{h+2}-\frac{1}{2t-h}\right)\\ & >0 \end{array}$$

When θ∈(0,π/4) and h≤t−2, we have $\cos\theta^{2t-1-h}\sin\theta^{h+1}>\sin\theta^{2t-1-h}\cos\theta^{h+1}$. Consequently, $f^{\prime }\left(\theta \right)>0$ holds. When θ∈(π/4,π/2) and h≤t−2, we have $\cos\theta^{2t-1-h}\sin\theta^{h+1}<\sin\theta^{2t-1-h}\cos\theta^{h+1}$ and $f^{\prime }\left(\theta \right)<0$. Moreover, note that θ=0,π/4,π/2 are extreme points of f(θ), it is obvious that $E\left[{\left(r_{1}\cos\theta +r_{2}\sin\theta \right)}^{2t}\right]$ reaches the maximum when θ=π/4. Thus, Lemma 1 follows. □

## Appendix C

**Proof** **of** **Lemma** **2.** Denote $v_{k}$ as the element of row $p_{r}$ and column $p_{c}$ in Φ, where k=1,2⋯K. By the definition of $\Phi_{K}$, we have $v_{k}=\sqrt{2}z_{\left(p_{c}-1\right)M+p_{r}}$. The left side of inequality (14) can be written as

$$\begin{array}{ll} E\left[Q_{m}^{2t}\right] & =E\left[{\left(v_{1}u_{1}+v_{2}u_{2}+\cdots v_{K}u_{K}\right)}^{2t}\right]\\ & =\sum_{R}E\left[{\left(v_{1}u_{1}+v_{2}u_{2}+R\right)}^{2t}\right]P\left(\sum_{k=3}^{K}v_{k}u_{k}=R\right) \end{array}$$

Using Lemma 1, we get

$$\begin{array}{ll} & \sum_{R}E\left[{\left(v_{1}u_{1}+v_{2}u_{2}+R\right)}^{2t}\right]P\left(\sum_{k=3}^{K}v_{k}u_{k}=R\right)\\ \leq & \sum_{R}E\left[{\left(v_{1}\sqrt{\left(u_{1}^{2}+u_{2}^{2}\right)/2}+v_{2}\sqrt{\left(u_{1}^{2}+u_{2}^{2}\right)/2}+R\right)}^{2t}\right]P\left(\sum_{k=3}^{K}v_{k}u_{k}=R\right) \end{array}$$

Let $\eta ={\left(\sqrt{\left(u_{1}^{2}+u_{2}^{2}\right)/2},\sqrt{\left(u_{1}^{2}+u_{2}^{2}\right)/2},u_{3}\cdots u_{K}\right)}^{T}$, we have

(A10) $$\begin{array}{ll} E\left[Q_{m}^{2t}\right] & \leq \sum_{R}E\left[{\left(v_{1}\sqrt{\left(u_{1}^{2}+u_{2}^{2}\right)/2}+v_{2}\sqrt{\left(u_{1}^{2}+u_{2}^{2}\right)/2}+R\right)}^{2t}\right]P\left(\sum_{k=3}^{K}v_{k}u_{k}=R\right)\\ & \;=E\left[{\left(\varphi_{m}^{T}\eta \right)}^{2t}\right] \end{array}$$

Applying this argument repeatedly yields Lemma 2, as η eventually becomes w [27]. Then, Lemma 2 holds. □

## Appendix D

**Proof** **of** **Lemma** **3.** Let ${\left\{t_{k}\right\}}_{k=1}^{K}$ be a group of i.i.d. variables and $t_{k}∼N\left(0,1\right)$, k=1,2⋯K. Rewrite T as $T=\frac{1}{\sqrt{K}}\sum_{k=1}^{K}t_{k}$. Substituting $\varphi_{m}^{T}\omega =\frac{1}{\sqrt{K}}\sum_{k=1}^{K}v_{k}$ into inequality (15), we obtain

$$\left\{ \begin{array}{l} E\left(T^{2t}\right)=\frac{1}{K^{t}}E\left[\sum_{n_{k}\geq 0,\sum_{k=1}^{K}n_{k}=2t}\left(\begin{array}{c} 2t\\ n_{1},n_{2}\cdots n_{K} \end{array}\right)\prod_{k=1}^{K}t_{k}^{n_{k}}\right]\\ E\left[{\left(\varphi_{m}^{T}w\right)}^{2t}\right]=\frac{1}{K^{t}}E\left[\sum_{n_{k}\geq 0,\sum_{k=1}^{K}n_{k}=2t}\left(\begin{array}{c} 2t\\ n_{1},n_{2}\cdots n_{K} \end{array}\right)\prod_{k=1}^{K}v_{k}^{n_{k}}\right] \end{array}\right.$$

where $\left(\begin{array}{c} 2t\\ n_{1},n_{2}\cdots n_{K} \end{array}\right)=\frac{\left(2t\right)!}{\left(n_{1}!\right)\left(n_{2}!\right)\cdots \left(n_{K}!\right)}$. When $n_{k}$ is odd, both $E\left(t_{k}^{n_{k}}\right)$ and $E\left(v_{k}^{n_{k}}\right)$ are equal to 0. When $n_{k}$ is even, let $n_{k}=2t$ and we get $E\left(t_{k}^{2t}\right)=\left(2t\right)!/\left(t!2^{t}\right)$ and $E\left(v_{k}^{2t}\right)=\left(2t\right)!/\left({\left(t!\right)}^{2}2^{t}\right)$. Obviously, $E\left(v_{k}^{n_{k}}\right)\leq E\left(t_{k}^{n_{k}}\right)$ holds. Therefore, Lemma 3 follows. □
